# Supplementary material for: HyperSLICE: HyperBand optimized spiral for low‐latency interactive cardiac examination
Source: Magn Reson Med. 2023 Oct 6;91(1):266–79. doi: 10.1002/mrm.29855 (PMC10953456; doi:10.1002/mrm.29855)
Supplement: Supplementary file 1 — Table S1. Nominal acquisition parameters for reference Cartesian breath‐hold, real‐time Cartesian, and optimized spiral trajectories acquired prospectively in patients. Table S2. The top three variable density spiral trajectories obtained from the HyperBand optimization. Figure S1. Trajectory optimization through HyperBand. Hyperparameters to generate spiral trajectories are updated depending on the resulting structural similarity (SSIM) scores obtained from deep artifact suppressed images. The trajectory shown in green corresponds to the resulting optimized trajectory (parameters found in Table 1). [file MRM-91-266-s004.docx]

**Supporting Information Document**

HyperSLICE: HyperBand optimised Spiral for Low-latency Interactive Cardiac Examination


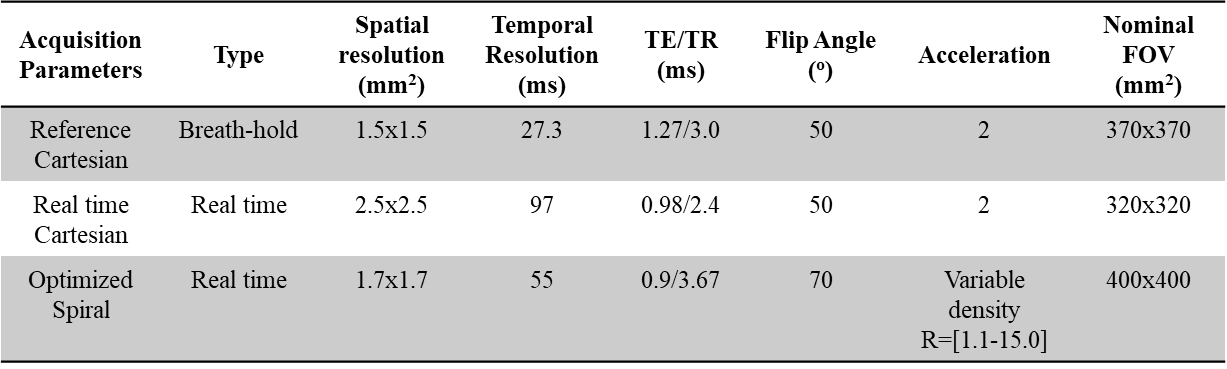


**Supporting Information Table S1.** Nominal acquisition parameters for reference Cartesian breath-hold, real-time Cartesian and optimized spiral trajectories acquired prospectively in patients.


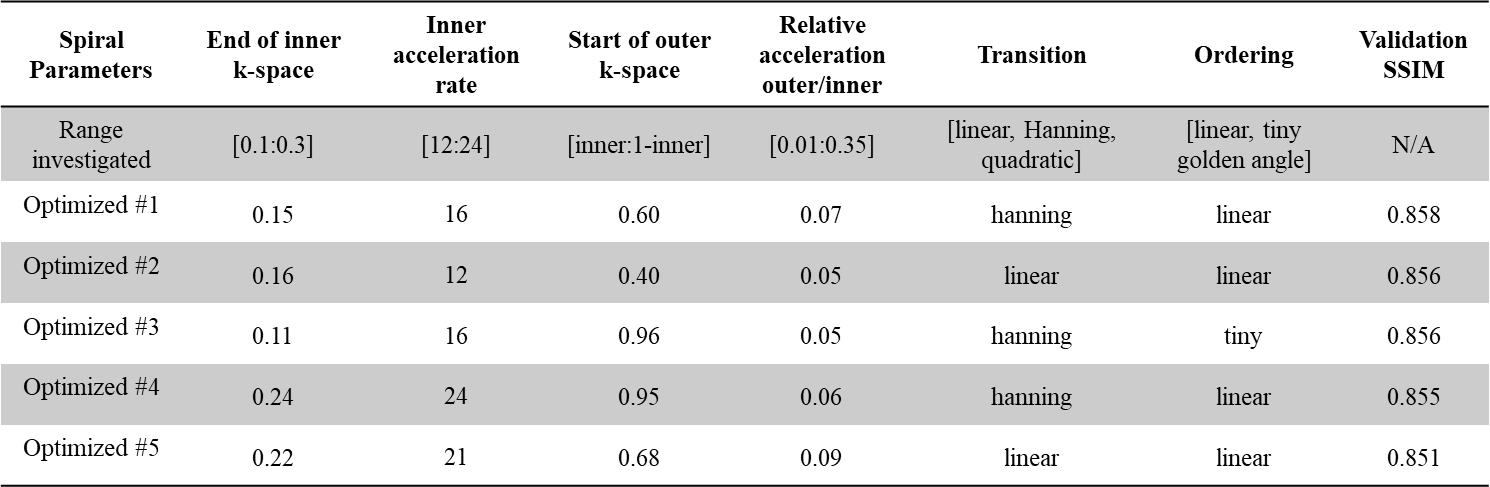


**Supporting Information Table S2.** The top three variable density spiral trajectories obtained from the HyperBand optimization.


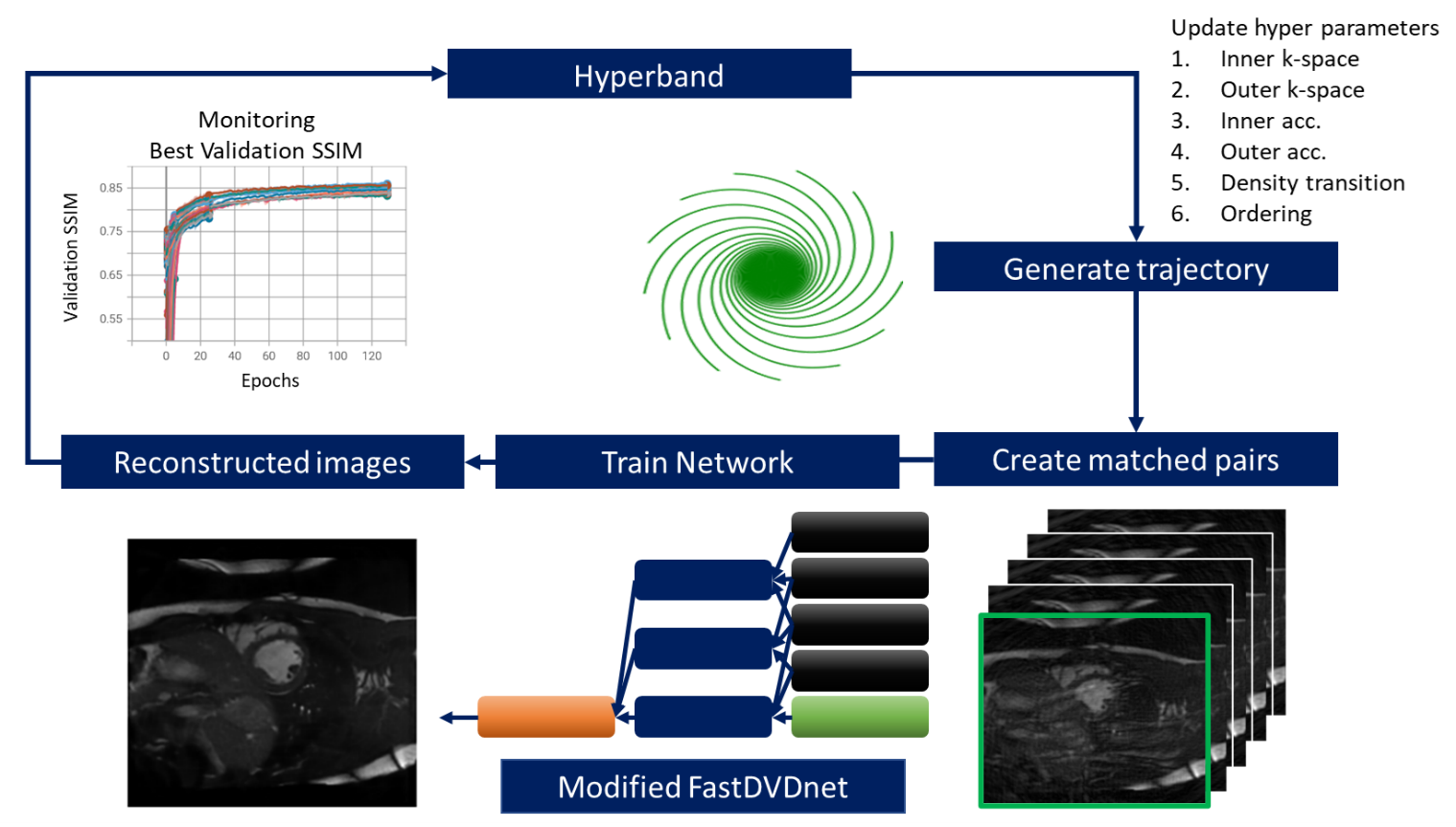


**Supporting Information Figure S1. Trajectory optimization through HyperBand:** Hyperparameters to generate spiral trajectories are updated depending on the resulting SSIM scores obtained from deep artifact suppressed images. The trajectory shown in green corresponds to the resulting optimized trajectory (parameters in Table 1).

**Supporting Information Video S1.** Interactive imaging as performed on the scanner during catheter pull-back. The interface allows the user to move the scan plane and images update with minimal latency enabling immediate feedback on the location of the catheter.

**Supporting Information Video S2.** Interactive imaging during catheter pull-back in a second patient with suspected pulmonary hypertension.

**Supporting Information Video S3.** Segment of the interactive scan during catheter pull-back reconstructed using all methods. The reconstructed segment had to be of a fixed orientation to compute coil sensitivities once for the comparison methods.

**Supporting Information Video S4.** SToRM, VarNet and HyperSLICE reconstructions of the same optimized spiral data acquired with different amounts of motion. From left to right: During breath-hold, free-breathing, exercise and peak exercise.

**Supporting Information Video S5.** HyperSLICE reconstructions using the same trained network of data acquired with: 1) the original FOV (400x400 mm^2^) and base resolution (240), 2) higher base resolution (288), 3) larger FOV (FOV - 450x450mm^2^) and 4) both changes (FOV - 450x450mm^2^, base resolution - 288).
